# Radiomics Analysis of Preprocedural CT Imaging for Outcome Prediction after Transjugular Intrahepatic Portosystemic Shunt Creation

**DOI:** 10.3390/life14060726

**Published:** 2024-06-03

**Authors:** Giuseppe Mamone, Albert Comelli, Giorgia Porrello, Mariapina Milazzo, Ambra Di Piazza, Alessandro Stefano, Viviana Benfante, Antonino Tuttolomondo, Gianvincenzo Sparacia, Luigi Maruzzelli, Roberto Miraglia

**Affiliations:** 1 Radiology Unit, IRCCS-ISMETT (Mediterranean Institute for Transplantation and Advanced Specialized Therapies), Via Tricomi 5, 90127 Palermo, Italy; mmilazzo@ismett.edu (M.M.); adipiazza@ismett.edu (A.D.P.); gsparacia@ismett.edu (G.S.); lmaruzzelli@ismett.edu (L.M.); rmiraglia@ismett.edu (R.M.); 2 Ri.MED Foundation, Via Bandiera 11, 90133 Palermo, Italy; acomelli@fondazionerimed.com (A.C.); vbenfante@fondazionerimed.com (V.B.); 3 Section of Radiology, Department of Biomedicine, Neuroscience and Advanced Diagnostics (Bi.N.D), University of Palermo, Via del Vespro 127, 90127 Palermo, Italy; giorgia.porrello@unipa.it; 4 Institute of Molecular Bioimaging and Physiology, National Research Council (IBFM-CNR), 90015 Cefalù, Italy; alessandro.stefano@ibfm.cnr.it; 5 Department of Health Promotion, Mother and Child Care, Internal Medicine and Medical Specialties, Molecular and Clinical Medicine, University of Palermo, 90127 Palermo, Italy; bruno.tuttolomondo@unipa.it

**Keywords:** radiomics, transjugular intrahepatic portosystemic shunt, computed tomography, recurrence, survival analysis, interventional radiology

## Abstract

Purpose: To evaluate the role of radiomics in preoperative outcome prediction in cirrhotic patients who underwent transjugular intrahepatic portosystemic shunt (TIPS) using “controlled expansion covered stents”. Materials and Methods: This retrospective institutional review board-approved study included cirrhotic patients undergoing TIPS with controlled expansion covered stent placement. From preoperative CT images, the whole liver was segmented into Volumes of Interest (VOIs) at the unenhanced and portal venous phase. Radiomics features were extracted, collected, and analyzed. Subsequently, receiver operating characteristic (ROC) curves were drawn to assess which features could predict patients’ outcomes. The endpoints studied were 6-month overall survival (OS), development of hepatic encephalopathy (HE), grade II or higher HE according to West Haven Criteria, and clinical response, defined as the absence of rebleeding or ascites. A radiomic model for outcome prediction was then designed. Results: A total of 76 consecutive cirrhotic patients undergoing TIPS creation were enrolled. The highest performances in terms of the area under the receiver operating characteristic curve (AUROC) were observed for the “clinical response” and “survival at 6 months” outcome with 0.755 and 0.767, at the unenhanced and portal venous phase, respectively. Specifically, on basal scans, accuracy, specificity, and sensitivity were 66.42%, 63.93%, and 73.75%, respectively. At the portal venous phase, an accuracy of 65.34%, a specificity of 62.38%, and a sensitivity of 74.00% were demonstrated. Conclusions: A pre-interventional machine learning-based CT radiomics algorithm could be useful in predicting survival and clinical response after TIPS creation in cirrhotic patients.

## 1. Introduction

Transjugular intrahepatic portosystemic shunt (TIPS) is a treatment of proven efficacy in cirrhotic patients that present with portal hypertension complications, such as variceal rebleeding or refractory ascites, which represent the main indications for TIPS creation. Other indications include refractory hepatic hydrothorax, and hepato-pulmonary, hepato-renal, or Budd–Chiari syndromes [1]. Despite its widespread use, one-year survival rates after TIPS creation still range from 48 to 90%, according to the severity of the underlying liver disease [1,2]. In TIPS patients, post-procedure liver failure, hepatic encephalopathy (HE), and sepsis are the most common determinants of poor prognosis [2]. The new controlled expansion covered stents (Viatorr Controlled Expansion Endoprosthesis, Gore, Newark, DE, USA) are expected to lower the incidence of HE and early liver failure, since these stents allow more accurate diameter control and may be customized during implantation, therefore reducing the risk of complications. However, HE and liver failure are still a concern after TIPS placement. A possible solution would be to preoperatively select the patients that potentially carry the lowest risk of complications.

With this aim, clinical scores have been developed, like the model of end-stage liver disease (MELD), which includes serum bilirubin, international normalized ratio (INR), and creatinine levels, as well as the presence of HE, pulmonary hypertension, and congestive heart failure [1]. The incidence of HE after TIPS placement is nonetheless still relatively high (ranging from 30% to 55%), even in patients without previous HE episodes or with mild liver disease [2].

New predictive models and biomarkers could be provided by artificial intelligence (AI), leading to better patient selection, avoiding TIPS whenever a poor outcome is expected. AI comprises radiomics, deep learning, and machine learning and has proven its value for the early diagnosis and treatment of various diseases [3,4], as well as in operator-independent liver segmentation on computed tomography (CT) [5].

Radiomics is a recent and promising field of research that, starting from simple medical images, allows the extraction and analysis of many quantitative features, converting them into meaningful data [3,4].

Current radiomics applications involve many aspects of hepatology, including prediction of cirrhosis and nonalcoholic fatty liver disease (NAFLD), liver fibrosis staging, and the differentiation between benign and malignant nodules [6,7,8]. Despite its potential clinical importance, to the best of our knowledge, machine learning-based CT radiomics models have not yet been implemented in patients undergoing TIPS, especially with new-generation controlled-expansion covered stents [9].

The aim of our study is to assess the feasibility of a radiomics model based on features extracted from routinely acquired CT images upon TIPS placement, so as to predict HE, overall clinical response, and survival after TIPS procedure.

## 2. Materials and Methods

### 2.1. Patients

All patients who underwent TIPS using controlled expansion covered stents, from May 2015 to June 2019 in our hospital, were retrospectively enrolled in this study, after searching their clinical records on our institutional electronical medical archive. Inclusion criteria were as follows: patients above 18 years old, with a defined diagnosis of cirrhosis, hospitalized for prophylaxis of variceal rebleeding, or refractory ascites; without malignant liver lesions or oncological history; presence of a good-quality CT study performed within 3 months upon the procedure; access to complete clinical medical records. Among exclusion criteria we considered: incomplete or low-quality imaging studies; incomplete clinical records; patients lost to follow-up; liver transplantation performed within 6 months from the procedure; presence of complete portal vein thrombosis and/or cavernomatous transformation of portal vein; hepatocellular carcinoma or other malignancies, and Budd–Chiari syndrome. Endpoints evaluated were 6-month survival, development of HE including pre-TIPS HE, grade II or higher grade HE according to West Haven Criteria (WHC) [10], and clinical response, defined as the absence of rebleeding or ascites.

This study followed the Helsinki declaration principles and was approved by the institutional review board of our hospital. Due to its retrospective nature, written informed consent was waived.

### 2.2. TIPS Procedure

All TIPSs were performed in an angiographic suite (Innova 4100, GE Medical Systems, Milwaukee, WI, USA) by two radiologists with over twenty years of experience in TIPS creation, with a technique already described in other publications [11,12]. All procedures were performed under general anesthesia with ultrasound guidance for portal vein targeting. In all patients, the new covered “controlled expansion stents” were used [11]. All patients were followed up on with clinical and Doppler Ultrasound evaluation at 1, 3, and then every 6 months after the procedure.

### 2.3. CT Examination

All CT studies were performed within one month before the procedure, using a 64-slice CT scanner (GE VCT 64 slice, GE, Milwaukee, USA). Images were stored in the local Picture Archiving and Communication System (PACS) of our hospital. Technical parameters applied were as follows: tube voltage 120 kV; tube current range 100–400 mA; gantry rotation time 0.5 s; slice thickness 2.5 mm; intervals 0 m; matrix 512 × 512; detector collimation of 64 × 0.625 mm; pitch 0.984: noise index 18; ASIR 30%; large body FOV. Pictures were acquired on craniocaudal direction, in respiratory apnea. The imaging protocol included an unenhanced scan followed by contrast-enhanced acquisitions at the arterial, portal venous, and delayed phases, acquired with a delay of 30, 65, and 180 s with bolus tracking, respectively. Iodinated contrast agent (Iopromide, Bayer Healthcare, Leverkusen, Germany) was injected with a dose 1.5 mL × Kg at a rate of 4.0 mL/s, with an automatic power injector.

### 2.4. Volume of Interest (VOI) Segmentation and Feature Extraction

Pre-TIPS CT studies were used instead of CT examinations after the TIPS procedure to evaluate the role of radiomics in predicting the outcomes and in preoperatively selecting patients. Furthermore, pre-TIPS CT scanning is usually performed to provide an evaluation of the liver vascular anatomy upon the procedure, while post-interventional follow-up is usually performed by color Doppler ultrasound (CDUS). CT images from the picture archiving and communication system (PACS) were reviewed blindly by two board-certified diagnostic radiologists with 21 and 16 years of experience in liver imaging. The liver analysis was performed drawing regions of interest (ROIs) along the whole hepatic profile slide by slide on all the CT axial images obtaining a whole liver volume, namely a VOI. We consider this method more accurate than the only one axial image at the level of the right portal vein shown in previous studies [9].

In an independent workstation (ADW 4.7, GE, Milwaukee, WI, USA), the two radiologists manually drew the ROIs in consensus, on both unenhanced and portal-venous-phase images, along the margins of the liver by excluding the large portal vessels in the hepatic hilum. This manual method is more accurate than the automatic segmentation technique [13,14,15]. Each ROI was delineated as close to the liver margin as possible but not beyond the hepatic margin to avoid the influence of adjacent organs and abdominal fat.

Once the target volume was obtained, features were extracted from VOIs through Pyradiomics 3.1.0 [16], an open-source piece of software by Image Biomarkers Standardization Initiative (IBSI) [17], a compliant analysis tool capable of automatically extracting radiomics features grouped into the following three classes: (1) morphological features describing geometrical characteristics of the VOI; (2) first-order statistical features, describing grey level distribution within the VOI; and (3) texture features describing grey level patterns within the VOI.

The interested readers are encouraged to refer to the website “https://pyradiomics.readthedocs.io/en/latest/features.html (accessed on 20 March 2024)” for a comprehensive description of the above-mentioned classes. Segmentation was performed on both unenhanced and portal-venous-phase images, to explore possible differences in radiomics results.

### 2.5. Feature Reduction and Selection

The algorithm used in this study for feature reduction and selection is a statistical system based on point-biserial correlation (pbc) and logistic regression analysis, as described by Barone et al. [18], and successfully used in many radiomics studied [19,20]. The goal of this algorithm is to categorize the features, assigning them a score, and then to select the most discriminant ones. Therefore, the pbc between each feature and the dichotomic outcome was calculated and used to sort the features in a descending fashion, according to the pbc score. Subsequently, a cycle was initialized, adding one feature at a time, and then performing a logistic regression analysis until the *p*-value increased. In this way, features with a valuable association that presented the desired outcome were identified and consequently used in the next step of this study, namely the implementation of the predictive model.

### 2.6. Predictive Model

To implement and evaluate the predictive model performance, the linear Discriminant Analysis (DA) was applied with the aim of overcoming the unbalanced dataset issue in our study. As a matter of fact, unbalanced datasets do not have a negative consequence on DA performance [21,22], as also shown in a study using DA in a radiomics model predicting hepatocellular carcinoma response after transarterial embolization [23]. Furthermore, the k-fold cross-validation strategy was employed by randomly dividing the dataset into k sub-datasets of equal size. This method yields more robust results compared to splitting data into ratios like 7:3 or 8:2. In the latter cases, variations in case distribution, coupled with the limited number of cases, could significantly alter performance. In essence, k-fold cross-validation mitigates overfitting and asymmetrical sampling, thereby enhancing result accuracy and robustness. Specifically, the dataset was divided into k-folds, with k = 5. The value 5 was determined empirically through a trial-and-error method (k range: 5–15, step size of 5). One of the folds was used as the test set and the remaining folds constituted the training set.

The k-fold strategy was used in such a way as to (1) guarantee disjointed test sets and (2) maintain the same percentage of patient status in the folds as in the original dataset. In this way, each time, a different fold, never used during training, was left for the test. This process was repeated five times, and the average performance across all experiments was computed.

### 2.7. Statistical Performance Analysis

The diagnostic performance was calculated using sensitivity, defined as the ratio between the number of samples correctly classified as positive and the number of true-positive (TP); specificity, defined as the number of samples correctly classified as negative divided by the number of true-negative (TN) samples; positive predictive value (PPV), defined as the degree to which repeated classifications under unchanged conditions show the same results; and accuracy. The obtained performance results were expressed as mean ± standard deviation.

Finally, the ability of the most significant radiomics features to predict the desired outcome was also assessed by means of receiver operating characteristics (ROCs) with 95% confidence intervals (CIs) and areas under the ROC curve (AUROCs).

## 3. Results

### 3.1. Study Population

A total of 130 consecutive patients who underwent TIPS procedure using controlled expansion stents from May 2015 to June 2019 were retrospectively included in this study. Among them, we excluded nine patients who underwent liver transplantation, one patient who presented with complete portal vein thrombosis, and one patient with Budd–Chiari syndrome. Twenty-three patients were also excluded since their last CT examination was performed > 3 months before the TIPS procedure. Furthermore, we excluded another 20 patients, who were lost to follow-up because we missed their 6-month survival data.

**Figure 1:**
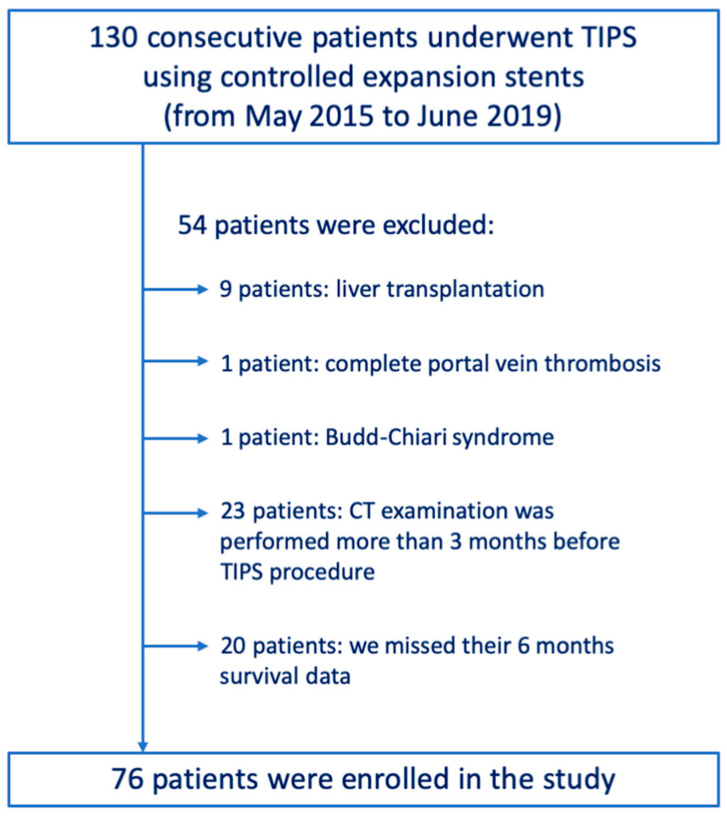
Selection of study population.

Finally, a total of 76 patients (Figure 1) were consecutively enrolled in this retrospective study (54 males and 22 females; mean age 59.2, standard deviation 8.9; median age 59.6; range 25–78 years). In this population, liver contrast-enhanced CT examinations were performed within 1, 2, and 3 months before the TIPS procedure in 42, 28, and 6 patients, respectively.

### 3.2. Clinical Characteristics

The etiology of chronic hepatic disease in the 76 patients analyzed was viral cirrhosis in 23 patients (30%), nonalcoholic steatohepatitis (NASH)-related cirrhosis in 22 patients (29%), alcohol-related in 24 patients (32%), cryptogenic in 3 patients (4%), biliary cirrhosis in 2 patients (3%), and cystic fibrosis in 2 patients (3%).

Clinical indications to the TIPS procedure were ascites in 51 patients (67%), bleeding in 20 patients (26%), and other causes (e.g., hydrothorax, portal vein thrombosis) in 5 patients (7%). Our population showed a MELD score ranging from 6 to 20.

After the TIPS procedure, overall overt HE was observed in 36 patients (47%). In this group, mild HE was present before TIPS placement in five cases (14%). This group of patients with overt HE observed after TIPS showed a pre–TIPS MELD score ranging from 6 to 20 (average: 11). Notably, six patients (8%) with HE before TIPS placement showed HE regression after the procedure. Overt grade II or higher HE according to West Haven Criteria was observed in 21 patients (28%), with a pre-TIPS meld score ranging from 7 to 17 (average: 11); in this group, only 3 patients (14%) had HE before the procedure.

At 6 months, the survival was 63 (83%) out of 76 patients, with a pre–TIPS MELD score ranging from 6 to 18 (average: 11). After the TIPS procedure, a clinical response (defined as the absence of rebleeding or ascites) was observed in 56 (74%) out of 76 patients, with a pre–TIPS MELD score ranging from 6 to 20 (average: 11). In all, 10 (13%) out of the 76 patients underwent liver transplantation.

During the total follow-up period, ranging from less than 1 month and 50.6 months after the TIPS procedure, 20 patients died (26%).

### 3.3. Performance of the Radiomics Model

For radiomics analysis, different outcomes were considered: (1) encephalopathy ≥ 2, (2) pre-TIPS encephalopathy, (3) overall encephalopathy, (4) clinical response, and (5) survival at 6 months. The number of patients and the relative percentage between positive and negative cases for each outcome are shown in Table 1.

Through Pyradiomics, 112 features were extracted from unenhanced and PVP CT images. The feature selection and reduction process were performed correlating features with different outcomes, as described in the previous “Feature reduction and selection” section. Table 2 shows the features identified for each outcome and for each type of CT study.

The discriminant features shown in Table 2 were then used to implement the DA-based predictive model.

Table 3 shows the diagnostic performance of the implemented predictive model for each outcome.

The AUROC ranged from 0.429 to 0.767 in unenhanced and portal-venous-phase CT studies, respectively.

The best performances we observed for the “clinical response” outcome with AUROC of 0.775 and 0.767, respectively, in unenhanced and portal-venous-phase images. Specifically, for the unenhanced CT study, the “clinical response” outcome showed an accuracy of 66.42% with a specificity of 63.93% and a sensitivity of 73.75%. For the portal-venous-phase CT study, the “clinical response” outcome showed an accuracy of 65.34%, a specificity of 62.38%, and a sensitivity of 74.00%.

We also observed a good performance for the “survival at 6 months” outcome in portal-venous-phase imaging, with an AUROC of 0.757, an accuracy of 71.23%, a specificity of 69.11%, and a sensitivity of 82.99%.

Furthermore, our predictive model showed a good performance for the “HE ≥ 2” outcome in unenhanced CT images, with an AUROC of 0.744, an accuracy of 73.21%, a specificity of 52.80%, and a sensitivity of 80.67%.

**Figure 2:**
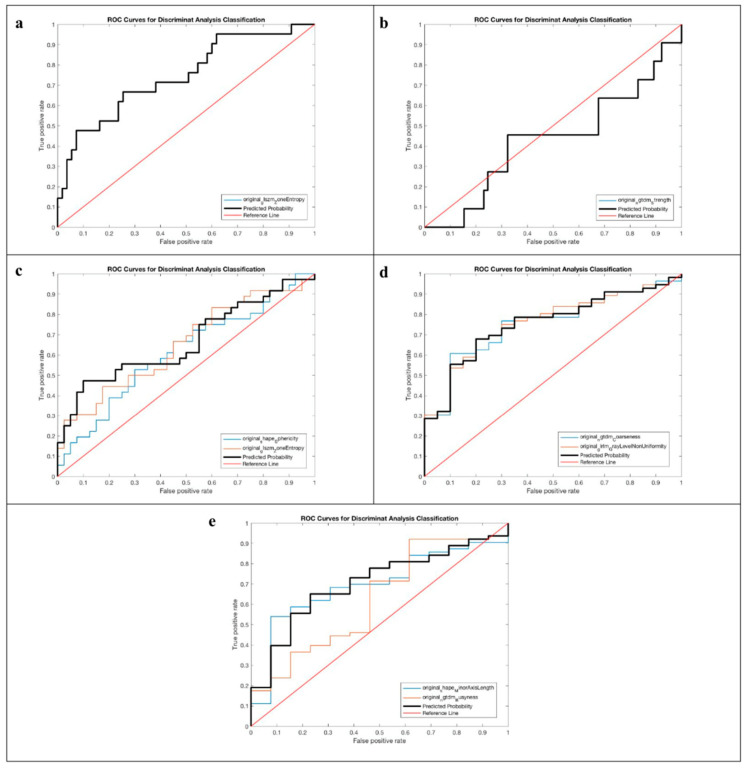
Receiver operating characteristic curves of the prediction model based on unenhanced CT radiomics features considering the following outcomes: (**a**) HE ≥ 2, (**b**) pre-TIPS HE, (**c**) HE, (**d**) clinical response, and (**e**) survival at 6 months. In the figures, the ROC curves for the predicted probability (black line) and the selected feature (blue line) coincide.

**Figure 3:**
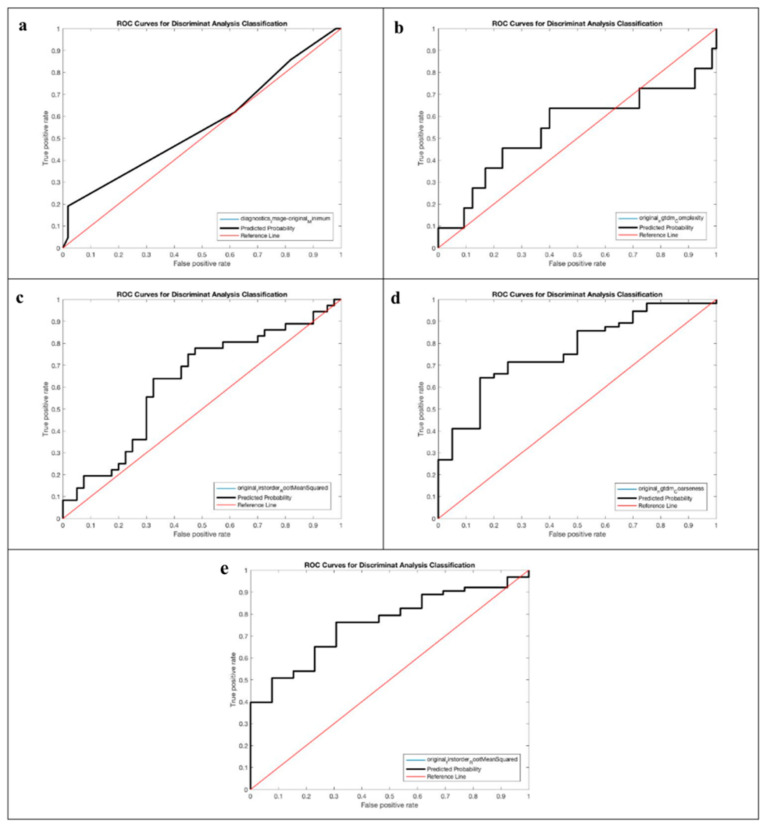
Receiver operating characteristic curves of the prediction model based on portal-venous-phase CT radiomics features considering the following outcomes: (**a**) HE ≥ 2, (**b**) pre-TIPS HE, (**c**) HE, (**d**) clinical response, and (**e**) survival at 6 months. In the figures, the ROC curves for the predicted probability (black line) and the selected feature (blue line) coincide.

For completeness of presentation, all ROC curves obtained for the unenhanced and portal-venous-phase CT studies are shown in Figure 2 and Figure 3, respectively.

## 4. Discussion

Careful selection of patients is of paramount importance to optimize the outcome of TIPS procedures. Nevertheless, no clinical parameters or scores (i.e., MELD scores) have been demonstrated to be effective outcome predictors in these patients [24,25]. In detail, although widely used to select patients before TIPS creation, it has been proven that MELD score calibration, the correspondence of observed to predicted mortality, is unsatisfactory with both the Mayo and UNOS MELD versions with C-statistics ranging from 0.66 to 0.72 [25]. It is, therefore, of paramount importance to recalibrate MELD scores and to look for new predictors or novel prediction models. In this scenario, our results suggest a possible role for radiomic analysis of preprocedural CT imaging that in our results was shown to be not inferior to actual MELD models in predicting procedural outcomes.

In this field, only one study has been recently published, by Cheng S et al. [9]. These authors demonstrated that a machine learning-based CT radiomics model had a better performance when compared to traditional clinical parameter-based models in the preoperative prediction of HE after TIPS procedures [9]. However, our study shows substantial differences from that of Cheng S et al. Mainly, (1) we explored various outcomes and not only a single outcome (HE); (2) in our population, the clinical indication to the TIPS procedure was mainly ascites (67% of patients) instead of bleeding, which represented the main indication in the study of Cheng S et al.; (3) our population included only patients undergoing TIPS procedures with the new-generation “controlled expansion stents”; (4) the liver analysis was performed drawing ROIs along the whole hepatic profile slide by slide on all the CT axial images obtaining a whole liver volume, while only one axial image at the level of the right portal vein was used in the analysis by Cheng S et al. [9].

Also, a study by Cao et al. [26] showed a potentially important role of radiomics, demonstrating that an integrated model of both radiomics and clinical features could perform well in predicting HE secondary to hepatitis B-related cirrhosis.

Our intent was to build a more comprehensive model using radiomics features for patient outcome prediction, other than presence of HE.

To overcome the issue of class imbalance that occurred in our dataset, among the many machine learning algorithms available at the present, we chose the DA. Indeed, a class imbalance issue might lead to unreliable results, since most machine learning approaches assume that the number of positive and negative cases is nearly equal. Rather than augmenting the data by artificially improving model performance, we chose the DA as a machine learning algorithm which is not very sensitive to unbalanced datasets [21,22].

Furthermore, the implemented k-fold strategy guaranteed disjointed test sets and maintained the same percentage of patient status in the different folds of the original dataset to increase the robustness of the results, unlike studies where models are trained on 80% of data and tested on the remaining 20% of data following the Pareto principle, also known as the 80/20 rule. It is important to note that, in these studies, the results strongly depend on how the choice of the test set is made, showing very optimistic performance in most cases. The results obtained encourage us to continue our efforts in a prospective study with a larger population, an external multicentric validation, and further exploration of alternative feature reduction and selection algorithms such as LASSO (Least Absolute Shrinkage and Selection Operator) or SVMs (Support Vector Machines) to identify additional predictive features that may not have been captured in this analysis. Furthermore, we will try to combine radiomics features with clinical factors, although the efficacy of combined models has shown no advantages over radiomics models alone [9].

Our study suggested that the proposed radiomics model based on routine CT examinations may be useful for predicting patients’ outcomes after the TIPS procedure. Specifically, a machine learning-based CT model for the prediction of different patients’ outcomes was implemented, namely presence of HE (including pre-TIPS HE), development of grade II or higher HE according to West Haven Criteria, clinical response, and survival at 6 months, with satisfying performance.

The highest performances of our radiomic model were observed for the “clinical response” outcome (absence of rebleeding or ascites) in unenhanced and portal-venous-phase images (AUROC of 0.755 and 0.767, respectively), for the “survival at 6 months” outcome in portal-venous-phase imaging (AUROC of 0.757), and for the “HE ≥2” outcome in unenhanced CT images (AUROC of 0.744), suggesting that radiomics could be implemented successfully for preoperative prediction of clinical response and survival of patients who need to undergo a TIPS procedure. This is an important notion that, especially if supported by other studies, could have paramount clinical implications for both radiologists and hepatologists. To our knowledge, this is the first study that tried to predict multiple outcomes in patients undergoing TIPS.

## Figures and Tables

**Table 1:** The five outcomes considered in the radiomics analysis (number of patients: 76).

Outcome	Positive	Negative
Hepatic Encephalopathy ≥2	21	55
Pre-TIPS Hepatic Encephalopathy	11	65
Hepatic Encephalopathy	36	40
Clinical response	56	20
Survival at 6 months	63	13

**Table 2:** Radiomics features identified for each outcome considering both unenhanced and portal-venous-phase CT images.

		Basal CT		Portal CT
Hepatic Encephalopathy ≥2	Feature	original_glszm_ZoneEntropy		diagnostics_Image-original_Minimum
	*p*-value	<0.001		0.008
pre-TIPS Hepatic Encephalopathy	Feature	original_ngtdm_Strength		original_ngtdm_Complexity
	*p*-value	0.081		0.036
Hepatic Encephalopathy	Feature	original_shape_Sphericity	original_glszm_ZoneEntropy	original_firstorder_RootMeanSquared
	*p*-value	0.026	0.020	0.023
Clinical response	Feature	original_ngtdm_Coarseness	original_glrlm_GrayLevelNonUniformity	original_ngtdm_Coarseness
	*p*-value	0.002	<0.001	<0.001
Survival at 6 month	Feature	original_shape_MinorAxisLength	original_ngtdm_Busyness	original_firstorder_RootMeanSquared
	*p*-value	0.050	0.020	0.013

**Table 3:** Diagnostic performance of the predictive model based on radiomics analysis for each outcome.

	Sensitivity [%]	Specificity [%]	PPV [%]	Accuracy [%]	AUROC	*p*-Value
	Basal CT					
Hepatic Encephalopathy ≥2	80.67	52.80	82.52	73.21	0.744 (0.615–0.874)	0.002
pre-TIPS Hepatic Encephalopathy	89.31	4.32	85.58	77.81	0.429 (0.229–0.629)	0.484
Hepatic Encephalopathy	66.75	51.11	61.13	59.44	0.664 (0.538–0.790)	0.038
Clinical response	73.75	63.93	42.77	66.42	0.755 (0.640–0.870)	<0.001
Survival at 6 month	76.53	58.15	25.93	61.21	0.704 (0.563–0.845)	0.017
	Portal CT					
Hepatic Encephalopathy ≥2	97.67	23.81	77.73	77.82	0.559 (0.385–0.733)	0.406
pre-TIPS Hepatic Encephalopathy	88.59	19.62	87.64	79.26	0.544 (0.312–0.775)	0.710
Hepatic Encephalopathy	73.08	50.32	62.66	62.45	0.627 (0.499–0.756)	0.044
Clinical response	74.00	62.38	41.71	65.34	0.767 (0.651–0.882)	<0.001
Survival at 6 month	82.99	69.11	33.78	71.23	0.757 (0.633–0.880)	<0.001

## Data Availability

The original contributions presented in the study are included in the article, further inquiries can be directed to the corresponding author.
